# Electroencephalogram-Based Single-Trial Detection of Language Expectation Violations in Listening to Speech

**DOI:** 10.3389/fncom.2019.00015

**Published:** 2019-03-29

**Authors:** Hiroki Tanaka, Hiroki Watanabe, Hayato Maki, Sakti Sakriani, Satoshi Nakamura

**Affiliations:** Division of Information Science, Nara Institute of Science and Technology, Nara, Japan

**Keywords:** electroencephalogram, event-related potentials, N400, P600, single-trial analysis, multilayer perceptron

## Abstract

We propose an approach for the detection of language expectation violations that occur in communication. We examined semantic and syntactic violations from electroencephalogram (EEG) when participants listened to spoken sentences. Previous studies have shown that such event-related potential (ERP) components as N400 and the late positivity (P600) are evoked in the auditory where semantic and syntactic anomalies occur. We used this knowledge to detect language expectation violation from single-trial EEGs by machine learning techniques. We recorded the brain activity of 18 participants while they listened to sentences that contained semantic and syntactic anomalies and identified the significant main effects of these anomalies in the ERP components. We also found that a multilayer perceptron achieved 59.5% (semantic) and 57.7% (syntactic) accuracies.

## Introduction

In speech communication, we often face several types of language expectation violations, such as prosodic, semantic, and syntactic errors, especially in conversation through machine output (e.g., human–computer interaction; Koponen, 2010). Questionnaire-based subjective judgments are commonly used to rate such language expectation violations as linguistic discrepancies (Dybkjær et al., 2007). For example, regarding errors in the responses of spoken dialogue systems and machine translation, human examiners in previous research judged each sentence on an error scale from 1 to 5, unlike automatic evaluation metrics, e.g., word error rate (Lippmann, 1997; Och et al., 1999; Papineni et al., 2002). Even though this approach is quick and practical, it suffers from several problems. For instance, such subjective evaluations of participants contain ambiguity and cannot guarantee accurate answers. In this paper, we propose a new objective approach that automatically detects such language expectation violations from physiological signals (Näätänen et al., 2004; Morikawa et al., 2011; Honda et al., 2018) because participants face more obstacles when they are manipulating physiological signals. Although our goal is to develop an online detection tool of the language expectation violations of humans using physiological signals, we simplify the problem by detecting clear language expectation violations as our first step. We assume that this system can also be used for assessing people who exhibit the anomalies of semantic context sensitivity (e.g., autism spectrum, dementia, Olichney et al., 2008; Pijnacker et al., 2010; O'Connor, 2012; Tanaka et al., 2012, 2015, 2017a,b, 2018a; Ujiro et al., 2018).

An electroencephalogram (EEG) is a non-invasive tool that records the electrical activity of the human brain with electrodes placed on the scalp. Regarding real applications using EEGs, in the context of motor imagery, which is reflected in event-related desynchronization [ERD; (Yeom and Sim, 2008)], the automatic detection of mental states based on convolutional neural networks (CNNs) has been proposed (Tang et al., 2017).

Unlike ERD, an event-related potential (ERP) is a measured time-locked brain response that is a direct result of a specific sensory, cognitive, or motor event. Since ERPs generally have a low signal/noise ratio in individual trials, many consecutive trials (e.g., 30 times) are usually averaged to diminish the random noise. Thus, single-trial detection of ERP components is very challenging due to their low signal/noise ratios (Blankertz et al., 2008; Lotte, 2015; Magee and Givigi, 2015). One public dataset focused on the single-trial detection of P300 components (Hald et al., 2006; Daubigney and Pietquin, 2011), which were elicited with relatively high signal/noise ratios. Most previous works have shown that P300 components can be detected with around 50–70% accuracy (exceeding the chance rate) using several machine learning algorithms (Stewart et al., 2014; Akram et al., 2015; Higashi et al., 2015; Sharma, 2017). Several approaches reached 100% accuracy using four to eight averaged trials in the BCI Competition 2003 (Cashero, 2012). We also need to consider that most works created subject-dependent models (within-subjects) because EEG signals are prone to being subject-dependent, and it remains challenging to generalize to subject-independent models (Terasawa et al., 2017).

Even though P300-based single-trial detection is one successful real application (P300-speller), it failed to detect language expectation violations including semantic and syntactic errors. To achieve single-trial detection of such errors, we focus on other ERP components, e.g., N400 and P600. N400 is a well-known ERP component that is evoked in auditory and visual modalities where semantic anomalies occur (Hagoort and Brown, 2000b). N400 is a phenomenon in which the potential shift in the negative direction increases around the brain's parietal region at around 400 ms from the onset of semantic and syntactic anomalies. Because N400 is strongly influenced by background noise, artifacts, and variations among trials, multiple times must be averaged. One study concluded that N400 is further influenced by a mismatch of the syntactic case information (Frisch and Schlesewsky, 2001). P600 (Narumi, 2014), another well-known ERP component (Hagoort and Brown, 2000a), is evoked in auditory and visual modalities where rule-governed anomalies generally occur. P600 is a language-related ERP that is thought to be elicited by grammatical errors and other syntactic anomalies. Several works have been done in Japanese (Ueno and Kluender, 2003; Mueller et al., 2007). P600 is characterized as a positive-going deflection with an onset around 500 ms after the onset of several types of anomalies. It peaks around 600 ms after the presentation of the stimulus and lasts several 100 ms. P600 is not language-specific, but it can be elicited in non-linguistic (but rule-governed) sequences [e.g., musical chords; (Patel et al., 1998)]. There are few P600 studies on Japanese syntactic violations in auditory modality (e.g., Mueller et al., 2005). To the best of our knowledge, no studies have addressed semantic violations in auditory modality in the Japanese language, which resemble our goal.

Based on our survey, despite the importance of real speech communication, only one study investigated the single-trial detection of semantic anomalies. Geuze et al. (2013) addressed the single-trial detection of semantic priming and the classification of visually presented related and unrelated words with an *L*_2_ regularized logistic regression algorithm as a classifier. For more practical applications with such technology, the work-detection keyboard autocorrection of possible semantic and syntactic errors from only EEGs identified the accuracy of the single-trial error detection of around 70% (Putze and Stuerzlinger, 2017). They used linear discriminant analysis as a classifier. Although these two studies detected semantic anomalies in single-trial levels, they did not detect them in spoken sentences.

In this paper, we propose the single-trial detection (from subjects who listened to spoken sentences) of semantic and syntactic anomalies that can be applied to Japanese spoken communication error evaluations. Such linguistic errors might be common across languages. Although we evaluated language expectation violations in Japanese, our approaches may be generalizable to other languages that include semantic (reflecting context expectation) and syntactic (reflecting rule-governed) errors. Understandably, when languages differ, the onset (starting points) of the time-locked ERPs will also be different.

This paper examined the following three research questions:

1. Do semantic violations while listening to spoken Japanese sentences elicit ERPs?
2. How does machine learning contribute to single-trial detection for language expectation violations, including semantic and syntactic errors?
3. Which classification model more proficiently distinguishes semantic and syntactic violations?

We recorded EEG data while Japanese participants listened to sentences that contained semantic and syntactic anomalies and analyzed the ERP effects. We also detected both anomalies from single-trial EEGs with a technique that classified them from multielectrodes and by integrating the time and spectral information with multiple machine learning algorithms.

This paper is an extension of conference proceedings (Tanaka et al., 2018b) in which we reported the overall single-trial detection of semantically incorrect sentences. We added the analysis of syntactic anomalies as well as participant-independent models with more participants.

## Methods

Our first aim is to confirm whether not only syntactic but also semantic violations in listening to Japanese sentences elicit ERPs. We hypothesized that semantic violations will elicit N400-/P600-related ERP components and syntactic violations will elicit P600-related ERP components. We also attempted to detect such violations from single-trial EEGs. We proposed several machine learning classifiers and confirmed classification above chance levels. In this section, we explain how we performed the EEG experiment and the classification.

### Participants

This study was carried out in accordance with the recommendations of the research ethical committee of the Nara Institute of Science and Technology. The protocol was approved by the research ethical committee of the Nara Institute of Science and Technology. All participants gave written informed consent in accordance with the Declaration of Helsinki.

Nineteen graduate students (16 males and 3 females) between 22 and 41 years of age (mean: 24.2) from the Nara Institute of Science and Technology participated. All were native Japanese speakers with no history of psychiatric problems or hearing disabilities; 18 were right-handed.

### Materials

In this study, we prepared two types of violations to elicit language expectation violations: a selectional restriction (as a semantic condition) and a double-nominative case (as a syntactic condition). Semantic violations very often also elicit biphasic N400 and P600 patterns, particularly when judging linguistic deviancy tasks (Sassenhagen et al., 2014). Note also that the double-nominative case violation that we chose for our syntactic manipulation has elicited N400 effects, including in Japanese (Mueller et al., 2005).

Japanese semantic and syntactic anomalies were manually created by referring to Takazawa et al. (2002) and Mueller et al. (2007). For the semantic condition, we defined error as a selectional restriction between a verb and its arguments. For the syntactic condition, error was defined a double-nominative case of the second phrase. We created an identical number of semantically and syntactically correct and incorrect sentences. We separated these sentences, which means that no two parts of the violated sentences are found in the stimuli.

The following is an example of two matched types of sentences (available on the Supplementary Material):


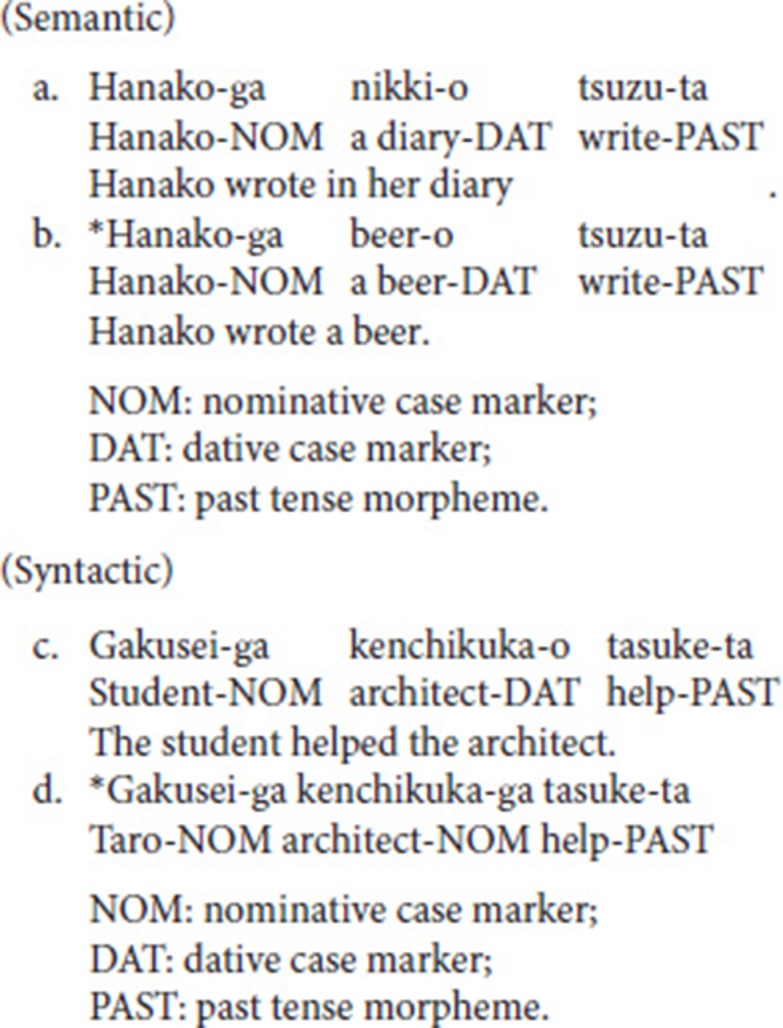


Here, an asterisk indicates semantically (b) and syntactically (d) incorrect sentences. Matched sentences corresponded in the first and third phrases. Due to the speech stimulus, we controlled the phonemes following Hagoort and Brown (2000b) in the third phrase to begin with plosive sounds:/t/,/k/,/d/, and/g/. Since such plosive sounds are in the onset position of the ERPs marked by human annotators, a consistent pattern is required in the spectrogram.

A group composed of the first author (A), the second author (B), and a graduate student who did not join our experiment (C) confirmed and corrected each sentence and reached a consensus about whether a semantic anomaly occurred. We selected the following 200 types of sentence from a total of 360 sentences: 40 semantically correct, 40 semantically incorrect, 40 syntactically correct, 40 syntactically incorrect, and 40 fillers sentences. Fillers were correct sentences that were used as dummies.

We transcribed them into text and recorded speech that was naturally spoken by a professional female narrator whom we instructed to avoid inserting pauses between phrases. The length of the audio files ranged from 1.8 to 3.0 s.

For the semantic case, the syntactic structure of the sentences was matched between the two conditions. We used the same target words in the third phrases. The experiment member A confirmed that the mean frequency of the third phrases was 1.02 in both conditions. Here, a mora is a unit in phonology that determines the syllable weight. The mean number of the moras of the third phrases was 4.25 (*SD* = 1.35). The difference of the two conditions was the second phrases with a mean number of moras of 4.15 (*SD* = 0.86) in the correct condition and 4.63 (SD = 0.93) in the incorrect condition.

For the syntactic case, the difference of the two conditions was the nominative case marker of the second phrases. The mean frequency of the second phrase was 1 in both conditions. The mean number of moras in the second phrases was 4.1 (SD = 0.98) in both conditions.

Moreover, we investigated the predictability of subsequent words (cloze probability) that affect the N400 amplitudes (Borovsky et al., 2010). One hundred crowdsourcing workers were given a list of 40 semantically incorrect sentences from which the final word had been removed. They read the sentences and filled in the blanks at the position of the hidden sentence-final words with the first word that popped into their heads. After that, we manually changed the present tense to the past tense, revised minor typing mistakes, and calculated the cloze probability of the most frequently selected words. The following is the distribution of the cloze probability: mean, 41%, SD, 16%, range, 14–85%. We confirmed that no words appeared as semantically incorrect in our stimuli, which means the cloze probability to the word is zero.

### Synchronization

Since ERPs are the time-locked brain response, we explain details with regard to synchronization between the auditory stimuli and EEG. Experiment members A and C marked the synchronized onset (*t* = 0). For the semantic case, ERP onset is the speech's start position of the third phrases. The onset starts with plosive sounds. The precise beginning position was marked by observing spectrogram of the speech. For the syntactic case, ERP onset is the speech's start position of the nominative case marker of the second phrases. The onset also begins with plosive sounds (only/g/) and was marked by observing spectrogram of the speech. We used the Wavesurfer (TMH, Speech, Music, and Hearing) in order to visualize spectrogram of the speech.

### Design

The participants entered a soundproof room, sat down, and were instructed to look at the attention point on the monitor and to refrain from blinking and moving as much as possible. The following was the experimental procedure: (1) watch the “+” mark for 1 s on the screen; (2) listen to one randomly presented speech sound for 4 s; and (3) press a key and determine within 2 s whether each speech contains grammatical or semantic errors. We conducted subjective evaluations and prepared practice trials before the EEG recordings. All these steps were completed within 25 min. For speech listening, we used earphones (ER1). This series of experiments was created using presentation software provided by Neurobehavioral Systems (Version 18.0, Neurobehavioral Systems, Inc., Berkeley, CA, www.neurobs.com).

The correct answer rates from the behavioral results were 95.8% for semantically correct and incorrect and 96.7% for syntactically correct and incorrect (error rate is <5%).

### Electroencephalogram Recording and Preprocessing

**Figure 1:**
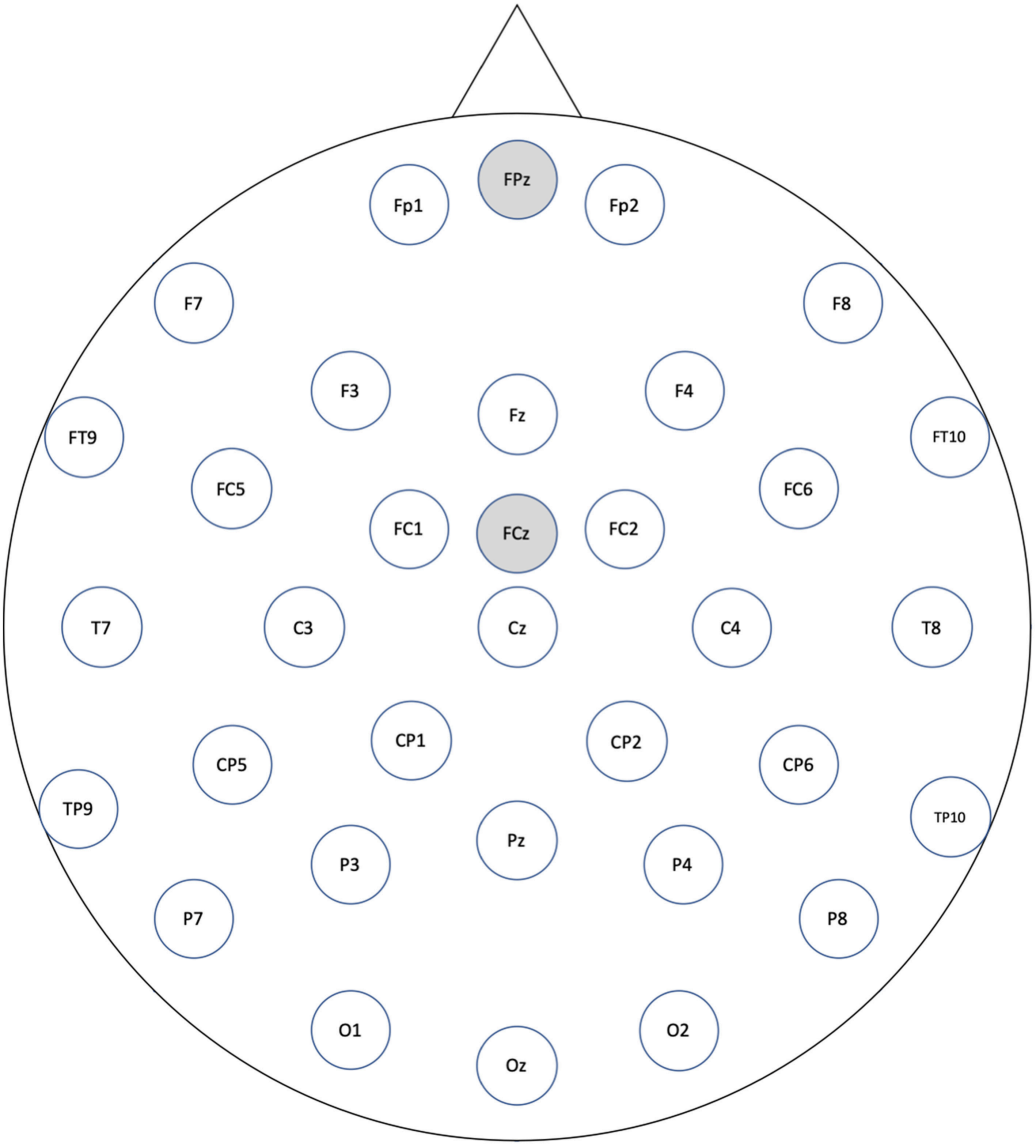
All electrode labels: gray electrodes indicate reference and grand position.

As an EEG cap, we used ActiCAP by Brain Products with 32 ch active electrodes according to all the standard positions of the international 10/20 system (see Figure 1). We used a BrainAmp DC from the same company as an amplifier. As a recording filter, we applied a high-pass filter of 0.016 Hz and a low-pass filter of 250 Hz. The sampling rate was 1,000 Hz, the reference electrode was FCz, and the ground electrode was FPz. In order to synchronize the speech signal with EEG, we generated a speech timing signal and recorded it with the EEG amplifier.

For preprocessing the recorded EEGs, we used FieldTrip software (Oostenveld et al., 2011) as follows: (1) Re-referencing was performed on the average of the TP9 and TP10 electrodes. (2) An FIRfilter was applied through a high-pass filter of 0.3 Hz (order: 6192), which is designed for DC suppression (−60 dB at DC) to replace the baseline correction (Maess et al., 2006; Wolff et al., 2008). (3) For each trial condition (excluding fillers), epochs were extracted at −100 to 900 ms of the synchronous onset. Here, the onset is the speech's start positions of the third phrases for the semantic condition and of the nominative case marker of the second phrases for the syntactic condition. (4) First artifact rejection was performed on epochs that exceeded a threshold of −350 and 350 μV in order to remove epochs contaminated with large amplitude of artifacts. This threshold rejection did not consider FP1 and FP2 electrodes where eye-related artifacts mainly contaminated. This large amplitude threshold is to preserve eye-blink artifact, which will be removed by later independent component analysis (ICA). (5) We performed an automatic approach and visual inspection to remove muscle artifacts: automatically identifying artifacts at *Z* score = 15 by considering amplitude distributions of band-pass-filtered epoch data (110–140 Hz), then rejecting epochs contaminated with muscle artifacts based on visual inspection (Meyer et al., 2017). (6) The recorded EEGs were downsampled to 250 Hz. (7) The logistic infomax ICA algorithm of Bell and Sejnowski (1995) was performed to correct eye-related artifacts, and eye-related components were removed. We identified the components by calculating the correlations to the FP1 and FP2 electrodes and by a visual inspection of the topographies and the waveforms. Four was the maximum number of rejected components because we only intended to remove as few horizontal and vertical ocular artifacts as possible. The rejected components had a mean of 2.1 (SD: 1.2). (8) A second artifact rejection was performed on epochs that exceeded the thresholds of −120 and 120 μV. As a result of the above artifact rejection procedures, one participant was removed because of the large number of rejected epochs (more than 30% of the epochs were rejected). The average rate of rejected trials across participants was 6.2%. We found no effects of the number of rejected trials between the semantically correct and incorrect and the syntactically correct and incorrect by using paired *t*-test {semantic: [*t*_(17)_ = 1.32, *p* = 0.20], syntactic: [*t*_(17)_ = 0.68, *p* = 0.51]}.

### Event-Related Potential Analysis

For further improvement of the signal/noise ratio, we applied another filtering procedure to the ERP data. Since the N400 components are around 6 Hz and the activity in the alpha frequency band tends to contaminate the EEG data, we used a two-pass IIR Butterworth filter of order 8 at 8 Hz to achieve a steeper frequency response than the FIR filter and to preserve the ERP components that also attenuate the alpha activity. Note that this filter was applied for only visualizing and analyzing ERPs, meaning that we did not use these filtered signals to the single-trial analysis.

We computed the grand average of all the participants. Based on a previous studies (Hagoort and Brown, 2000a,b; Mueller et al., 2005; Wolff et al., 2008), we selected the following electrodes in each time window: 100–300, 300–500, and 500–800 ms. These time windows were selected based on the previous study that analyzed syntax- and semantic-related ERP effects (Mueller et al., 2005). To assess the topographic differences in the ERPs, electrodes were summed up in five regions of interest (ROIs)—left anterior: F3, F7, FC1, FC5; right anterior: F4, F8, FC2, FC6; left posterior: CP1, CP5, P3, P7; right posterior: CP2, CP6, P4, P8; and midline: Fz, FCz, Cz, Pz. For the statistical analyses, we calculated the mean amplitudes in the chosen time windows (Wolff et al., 2008).

We used two-way repeated ANOVAs to examine the main effects of the condition and its interaction by ROIs in each time window. We performed a *post hoc* multiple comparison of the interaction between conditions and regions using the Tukey–Kramer method. Finally, we performed cluster-based permutation tests (Maris and Oostenveld, 2007) on the ERPs of the semantic and the syntactic conditions. Regarding the cluster-based permutation tests, for each time step of interest, we marked the electrodes that are members of significant clusters. The significance probability can be calculated by means of the Monte Carlo method. The Monte Carlo significance probability is also called a *p*-value. If the *p*-value is smaller than the critical alpha level (5% in this study), then we conclude that the data in the two experimental conditions are significantly different. Overall, we set the significance level to 5%.

### Feature and Classifiers

Based on previous work (Hagoort and Brown, 2000b; Roehm et al., 2004), we extracted the average values of the 100–300, 300–500, and 500–800 ms amplitudes from all of the electrodes (93 time domain features). To avoid overfitting to the training data, we selected specific time domains (possibly important time ranges) rather than using all time sampling points (simplifying the model). We also considered all of the electrodes with frequency domains for the single-trial detection of EEGs (Putze and Stuerzlinger, 2017). The delta band has been associated with N400 and P600 components in language (Correia et al., 2015). Thus, we performed a fast Fourier transform on the waveform between 0 and 900 ms to the onset and calculated the average values of the power spectra of δ (1–3 Hz), θ (4–7 Hz), α (8–12 Hz), and β (13–28 Hz) (124 spectral domain features) by referring to previous work (Hald et al., 2006; Mcmahon et al., 2015). We concatenated time and spectral features (217 dimensions). The feature vectors were normalized to a mean of zero and one standard deviation.

For the classifiers, we used a linear kernel support vector machine (L-SVM), a radial kernel support vector machine (R-SVM), a random forest (RF), and multilayer perceptrons (MLPs). The classifiers were trained on a dataset that combined 13 participants and subsequently tested on five different participants without further training by following Vareka and Mautner (2017). We observed how our detection models performed when they dealt with data from previously unknown participants.

These models were trained using 5-fold cross-validation for hyperparameter tuning on the training set to optimize the accuracies. The hyperparameters included the kernel (linear or radial basis function), *C* = {10^–5^, 10^–4^, …, 10^3^}, γ = {0.00, 0.005, …, 1.00} (in the case of the RBFkernel) for the SVMs, the number of variables tried at each split = {5, 10, 15, 20} for the RF, and the number of hidden units {5, 10, 50, 100, 150, 200}, the number of hidden layers {1, 2, 3}, and activation function (logistic, hyperbolic tangent, or rectified linear unit) in the MLP by referring to Vail et al. (2018). After the parameters were found, the models were trained on the whole training dataset and subsequently tested.

By a binomial test, we compared the chance rate (50.4% for the semantic sentences and 50.4% for the syntactic sentences in the test set) and the model that achieved the highest accuracy as well as precision, recall, and F1. We also calculated the correlation between cloze probability and semantic accuracy based on Pearson's correlation coefficient.

## Results

### Event-Related Potential Effects

**Figure 2:**
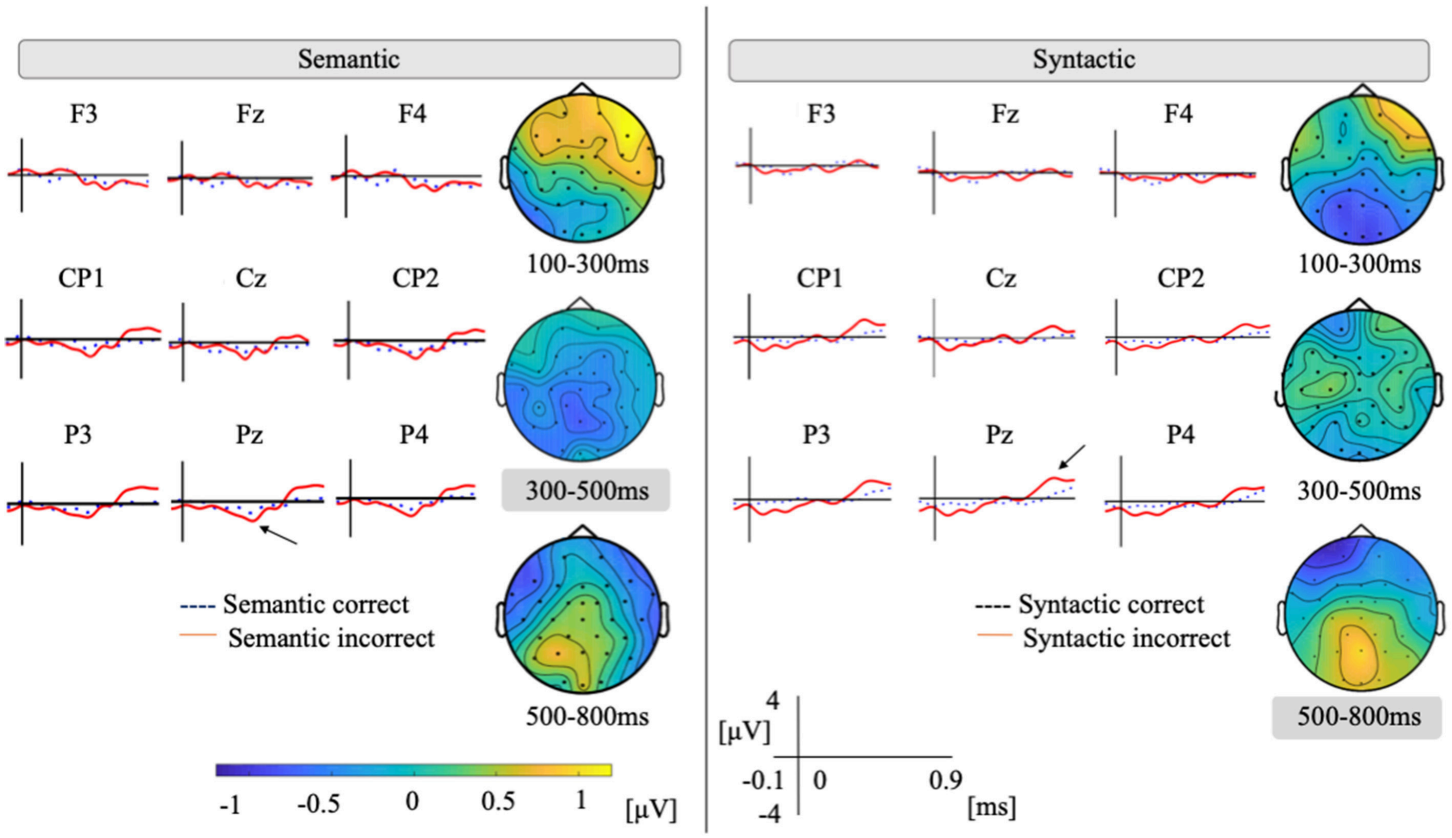
Grand average of nine representative electrodes of semantic and syntactic conditions. Vertical axis ranges between −4 and +4. Positivity is plotted up. We plotted −100 to 900 ms to the stimuli onset. Topographical map shows a difference wave between incorrect and correct conditions.

Figure 2 plots the ground averages at representative electrodes in the semantic and syntactic conditions. For the semantic condition, a potential shift to the negative around 400 ms can be observed under the semantically incorrect condition over the parietal region, and late positivity (P600) can also be seen.

Based on our assumption, for a time window of 300–500 ms, ANOVAs would show the main effects of the condition [*F*_(1, 17)_ = 4.69, *p* = 0.04]. No significant interaction was shown between condition by region [*F*_(4, 68)_ = 1.18, *p* = 0.32]. Regarding other time windows, for a mean amplitude of 100–300 ms, we found main effects of condition [*F*_(1, 17)_ = 4.51, *p* = 0.04] and also a significant interaction of condition by region [*F*_(4, 68)_ = 11.5, *p* < 0.001]. Since there were significant interactions of the condition by region, multiple comparisons were separately calculated for each region. *Post hoc* analysis by the Tukey–Kramer method revealed that the left anterior [difference (incorrect – correct): 0.66, *p* = 0.02, 95% CI = 0.09–1.23] and the right posterior (difference: 0.45, *p* = 0.02, 95% CI = 0.06–0.83) were significantly different between two conditions. For the mean amplitude of 500–800 ms, we found no main effects of condition [*F*_(1, 17)_ = 0.82, *p* = 0.37]. However, we did identify a significant interaction of condition by region [*F*_(4, 68)_ = 5.39, *p* < 0.001]. *Post hoc* analysis revealed that the left posterior (difference: 0.54, *p* = 0.008, 95% CI = 0.003–1.01) and the right anterior (difference: 0.88, *p* < 0.001, 95% CI = 0.51–1.2) were significantly different between two conditions.

For the syntactic condition, we observed a potential shift to the positive after 500 ms under the syntactically incorrect condition over the parietal region. Based on our assumption, for the time window of 500–800 ms, ANOVAs showed no main effects of condition [*F*_(1, 17)_ = 1.00, *p* = 0.33]. ANOVAs showed the interaction of the condition by region [*F*_(4, 68)_ = 6.03, *p* < 0.001]. *Post hoc* analysis revealed that the left posterior (difference: 0.51 μV, *p* = 0.04, 95% CI = 0.003–1.01 μV) and the right posterior (difference: 0.45 μV, *p* = 0.02, 95% CI = 0.06–0.83 μV) were significantly different between two conditions. Regarding other time windows, for the mean amplitude of 100–300 ms, we found no main effects of condition [*F*_(1, 17)_ = 1.28, *p* = 0.27]. However, we did find a significant interaction of the condition by region [*F*_(4, 68)_ = 6.86, *p* < 0.001]. *Post hoc* analysis revealed that the left posterior (difference: −0.65 μV, *p* = 0.006, 95% CI = −1.08 to −0.21 μV) and the right anterior (difference: −0.49 μV, *p* = 0.02, 95% CI = −0.90 to −0.08 μV) were significantly different between two conditions. For the mean amplitude of 300–500 ms, there were no main effects of condition [*F*_(1, 17)_ = 0.05, *p* = 0.82] and no interaction of the condition by region [*F*_(4, 68)_ = 0.05, *p* = 0.79].

**Figure 3:**
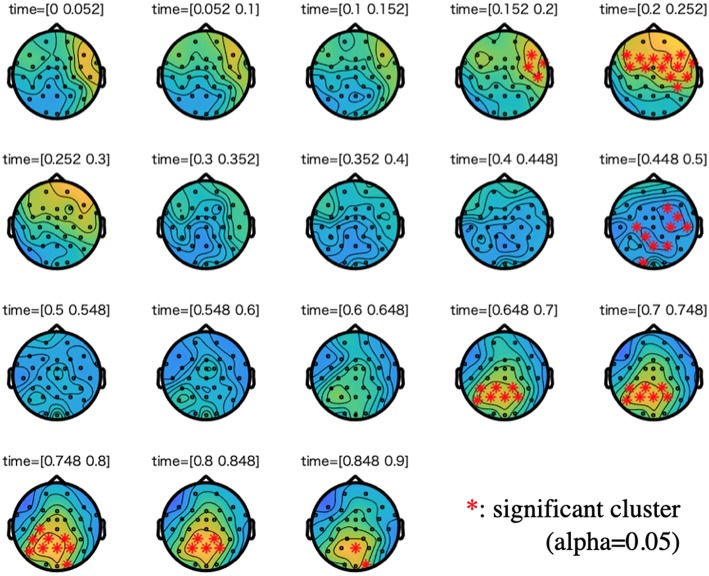
Cluster-based permutation tests on the event-related potentials (ERPs) of the semantic condition along with a difference wave between incorrect and correct conditions. We plotted 0–900 ms to the stimuli onset. For each time step of interest (time range: 0.05), we highlighted the electrodes that are members of significant clusters (cluster alpha value: 0.05). A cluster is significant if its *p*-value is less than the critical alpha level.

**Figure 4:**
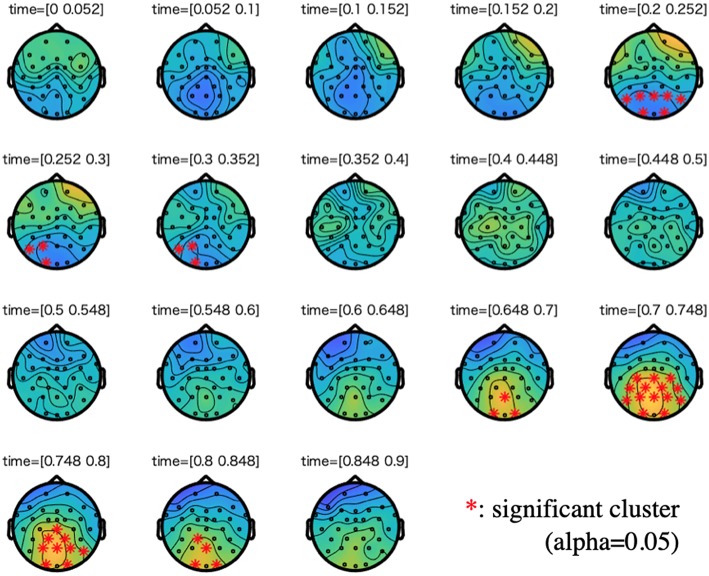
Cluster-based permutation tests on the ERPs of the syntactic condition along with a difference wave between incorrect and correct conditions. We plotted 0 to 900 ms to the stimuli onset. For each time step of interest (time range: 0.05), we highlighted the electrodes that are members of significant clusters (cluster alpha value: 0.05). A cluster is significant if its *p*-value is less than the critical alpha level.

Figures 3, 4 show the results of cluster-based permutation tests on ERPs of the semantic and the syntactic conditions.

### Single-Trial Detection

Table 1 indicates the accuracy of each classifier in the test sets. For the semantic conditions, MLP achieved the highest accuracy of 59.5%. Regarding this accuracy, we confirmed a statistical significance compared to the chance rate (*p* < 0.05): 44.3% precision, 63.1% recall, and 52.1% F1.

**Table 1:** Unweighted accuracies (%) of classifiers.

**Violations**	**L-SVM**	**R-SVM**	**RF**	**MLP**
Semantic	58.2	56.0	58.2	**59.5**
Syntactic	54.7	54.7	55.3	**57.7**

*The best model is indicated in bold*.

We found no significant correlation between the cloze probability or the predicted accuracy in the semantic condition (all classifiers, *r* < 0.15, *p* > 0.05).

For the syntactic conditions, the highest accuracy was also found when using MLP (57.7%), and we confirmed a statistical significance compared to the chance rate (*p* < 0.05): 58.8% precision, 57.9% recall, and 58.4% F1.

## Discussion

The aim of the present study is to observe the time-locked effects of semantic and syntactic anomalies in spoken Japanese sentences and to detect them with single-trial EEGs. We achieved this by focusing on the previous approach: ERPs. We followed two previous studies that elicited the ERP components of N400 and P600 in Japanese: Mueller et al. (2007) and Takazawa et al. (2002). We hypothesized that semantic violations will elicit N400-/P600-related ERP components and syntactic violations will elicit P600-related ERP components. We also attempted to use SVMs, RF, and MLP for single-trial EEGs and confirmed classification that exceeded chance levels. We next summarize our discussion regarding ERP analysis and single-trial detection.

### Event-Related Potential Analysis

For the semantic condition, we used such previously proposed stimuli as selectional restriction (Takazawa et al., 2002). Although the previous study was performed with visual stimuli, our experiment confirmed that ERP components were elicited even in an auditory experimental design.

One of our experiment's drawbacks is that semantically incorrect sentences were limited to the anomalies of the selectional restrictions at the end of sentences. Our 40-filler setting is limited to natural settings, and naturalistic sentence processing is a major analysis challenge. We identified several participants who did not indicate the strong effects of ERPs. We need to control such related factors as social traits and the attention of the participants as well as age (Constantino and Gruber, 2012).

Onset is another critical aspect for analyzing ERPs. We set the ERP onset to the speech's start position of the third phrases for the semantic condition and the speech's start position of the nominative case marker of the second phrases for the syntactic condition. Because this study uses auditory stimuli (speech sequences), we did not know the actual timing when the participants perceived the violations. In the future, we will measure the effects in the onset latency of a representative range of ERPs and implement artificial time shifting (Kiesel et al., 2008; Zoumpoulaki et al., 2013; Sassenhagen et al., 2014).

### Single-Trial Detection

Our classification model achieved 59.5% (semantic) and 57.7% (syntactic) detection accuracies in the incorrect conditions and outperformed the chance rate. MLP outperformed the other classifiers: SVMs and RF. Such accuracies were similar or superior to previous related works (Geuze et al., 2013; Higashi et al., 2015; Putze and Stuerzlinger, 2017). The previous work that detected semantic priming with 12 subjects showed accuracy between 51 and 63%, which is above chance in a cross-subject study (Geuze et al., 2013). Although our evaluation was validated by previously unseen participants, the MLP achieved a similar accuracy.

The N400 amplitude for incongruent words was also modulated by the cloze probability of the expected congruent word for that place. Generally, the best predictor of a word's N400 amplitude in a given sentence is its cloze probability (Kutas and Hillyard, 1984). The N400 amplitude is largest for items with low cloze probability and smallest for items with high cloze probability. Semantic anomaly thus shows the end point on a continuum of expectedness in a particular context (Coulson, 2001). Thus, we hypothesized that detecting low cloze probability items (large N400 amplitude) is easier because of the relatively high signal/noise ratios (Hald et al., 2006; Daubigney and Pietquin, 2011). However, we did not find a relationship between accuracy and cloze probability. This is because we did not control the cloze probability of the semantic incorrect sentences or the semantic correct sentences prior to the experiment (Borovsky et al., 2010).

This study did not consider the effects of the individuality of the frequency band. We fixed the frequency bands rather than individually adapting them based on individual alpha frequencies. This idea needs to be considered due to the high individual variability in this domain (Klimesch, 2012).

To improve classification accuracy, we need to increase the sophistication of the machine learning models, although EEGs have a low signal/noise ratio. We believe that a participant-adaptive technique (e.g., maximum likelihood linear regression; Gales and Woodland, 1996; Pan and Yang, 2010) is one possible future direction. Due to a large amount of P300 data, such as for a BCI competition, we applied several types of machine learning approach to our collected data by transfer learning (Pan et al., 2016).

Another possible direction to improve the classification accuracy is to average several trials (not a single trial) whose usefulness has already been validated. Several approaches achieved 100% accuracy using only four to eight averaged trials on P300 data (Cashero, 2012). We can apply this approach to detect the language expectation violations toward practical usage.

We will also improve our model using graph regularized tensor factorization (Maki et al., 2018) as well as non-negative matrix factorization, which we previously proposed. Automatic onset detection and the techniques of artificial shifted trials are also needed for completely automated anomaly detection (Kutas and Hillyard, 1980).

## Conclusions

This study aims to detect semantic and syntactic anomalies from a one-shot EEG, using a machine learning technique. We measured the EEGs of 18 participants while they listened to semantically anomalous sentences and confirmed N400- and P600-related ERP components. When using MLP, we achieved detection accuracies of 59.5% (semantic) and 57.7% (syntactic) with time and spectral domain inputs. From here, the results suggest that machine learning might be able to detect semantic and syntactic anomalies from correct sentences.

## Data Availability

The datasets for this study will not be made publicly available because of the Act on the Protection of Personal Information.

## Author Contributions

HT, HW, and HM performed the experiments and data analysis and conceived the methodology and the machine learning algorithms. HT and HW performed EEG preprocessing. SS and SN conceived the entire experiment design and analyzed, and discussed the results. HT wrote this manuscript. All of the authors reviewed the manuscript.

### Conflict of Interest Statement

The authors declare that the research was conducted in the absence of any commercial or financial relationships that could be construed as a potential conflict of interest.
